# Simvastatin and fluvastatin attenuate trauma-induced cell death and catabolism in human cartilage

**DOI:** 10.3389/fbioe.2022.965302

**Published:** 2022-09-09

**Authors:** Jana Riegger, Svenja Maurer, Sai Pulasani, Rolf E. Brenner

**Affiliations:** Department of Orthopedics, Division for Biochemistry of Joint and Connective Tissue Diseases, Ulm University, Ulm, Germany

**Keywords:** simvastatin, fluvastatin, statin therapy, cartilage, osteoarthritis, anti-catabolic, antioxidative, cell protective

## Abstract

Joint injuries are known to induce pathomechanisms that might lead to posttraumatic osteoarthritis (PTOA). In this regard, statins with their pleiotropic effects could represent potential therapeutic agents in preventing the development of PTOA. Therefore, we investigated the effects of simvastatin and fluvastatin in a drop-tower-based human *ex vivo* cartilage trauma model. After 7 days, a mechanical impact (0.59 J) resulted in a decrease of the cell viability and increased expression of catabolic enzymes in cartilage explants. Simvastatin and fluvastatin treatment of impacted cartilage demonstrated cell protective effects in a concentration dependent manner. Moreover, statin therapy exhibited chondroprotective effects as demonstrated by attenuated expression of MMP-2 and MMP-13 as well as subsequent breakdown of collagen type II (after impact). Further analysis indicated antioxidative properties of the statins by upregulating the gene expression of SOD2 and suppression that of NOX2 and NOX4. Despite its protective effects, simvastatin impaired the biosynthesis of collagen type II, which was confirmed during chondrogenic redifferentiation of high passage chondrocytes. However, while long-term administration of statins for 4 weeks impaired chondrogenic redifferentiation, addition of simvastatin at low concentrations for 1 week exhibited a slightly promoting effect. In conclusion, our data imply that simvastatin and fluvastatin are suitable in terms of initial harm reduction after cartilage trauma.

## 1 Introduction

Statins are potent hydroxymethylglutaryl-coenzyme A (HMG-CoA) inhibitors, commonly used as cholesterol-lowering drugs in hypercholesterolemia and cardiovascular disease. Moreover, statins have been found to possess pleiotropic therapeutic effects, comprising anti-inflammatory (Jain and Ridker, 2005) and antioxidative (Franzoni et al., 2003) potential, and have different impact on cell viability, ranging from pro-apoptotic to cell protective (Wood et al., 2013). Due to its anti-inflammatory properties, which are mainly based upon immunomodulation, including inhibition of leukocyte-endothelial adhesion and leukocyte recruitment (Obama et al., 2004; Weitz-Schmidt et al., 2001; Rezaie-Majd et al., 2002), statins have been considered as potential therapeutics in autoimmune diseases such as rheumatoid arthritis (RA) (Greenwood et al., 2006; McCarey et al., 2004; Leung et al., 2003). Clinical studies also imply beneficial effects of statin administration in patients suffering from osteoarthritis (OA) (Veronese et al., 2019; Kadam et al., 2013), the most common joint disorder among the elderly population, which is usually characterized by considerable lower inflammation levels as compared to RA (Robinson et al., 2017; Livshits and Kalinkovich, 2018).

The pathogenesis of OA is very complex and has not been completely understood so far. However, mechanical trauma has been identified as a crucial trigger of OA-related pathomechanisms, comprising enhanced oxidative stress and catabolism, immediate and ongoing cell death, synovial inflammation and impairment of chondroanabolic processes (Riegger and Brenner, 2020). In sum, the surviving chondrocytes undergo a phenotypical alteration, characterized by excessive release of catabolic enzymes, such as matrix metalloproteinases (MMPs) and a disintegrin and metalloproteinase with thrombospondin motifs (ADAMTS) as well as pro-inflammatory chemokines, a detrimental expression pattern which has also been associated to cellular senescence (McCulloch et al., 2017). Trauma-induced pathomechanisms can persist over years, driving progressive cartilage degeneration and leading to the development of a posttraumatic OA (PTOA). Therapeutic application of different statins, i.e. mevastatin and simvastatin, have been found to reduce injury-associated development of OA in animal models by reducing synovial inflammation after surgical destabilization of the joint (Akasaki et al., 2009; Aktas et al., 2011). However, only little is known about the direct effects of statins on chondrocyte death and cartilage metabolism after traumatic impact. Therefore, the present study primarily focuses on potential therapeutic effects of fluvastatin and simvastatin under low-grade inflammatory conditions in a human *ex vivo* cartilage trauma model, considering cell survival and catabolic processes.

Moreover, controversial results regarding the influence of statins on the expression of extracellular matrix (ECM) components, i.e. collagen type II and aggrecan, in isolated chondrocytes (Fafilek et al., 2017; Hatano et al., 2003; Han and Kim, 2016) as well as during chondrogenic differentiation of mesenchymal stem cells (MSC) (Zhang et al., 2019; Izadpanah et al., 2015) raised questions about the chondroanabolic potential of statin administration. Since both early harm reduction as well as stabilization of the chondrogenic phenotype of surviving chondrocytes might be decisive for the recovery of cartilage homeostasis after traumatic injuries, impact of statins on re-differentiation of high passage chondrocytes was additionally addressed in our study.

## 2 Materials and methods

### 2.1 Cartilage explants preparation and cultivation conditions

In this study human cartilage was obtained with written permission of donors undergoing total knee joint replacement due to OA and used according to the guidelines of the Declaration of Helsinki and of the Ethical Committee of the University of Ulm, Germany (ethical approval number: 353/18).

Overall, macroscopically intact tissue samples (International Cartilage Repair Society score ≤1) from femoral condyles were included in the study. Full-thickness cartilage explants (Ø = 6 mm) of 6 patients (range 61–85, mean age 72) were harvested, weighted and cultivated in serum-containing chondrocyte medium [[1:1] Dulbecco’s Modified Eagle’s Medium (DMEM)/Ham’s F12, 10% heat-inactivated fetal bovine serum, 0.5% penicillin/streptomycin (PAA Laboratories, Pasching, Austria), 0.5% L-glutamine and 10 μg/ml 2-phospho-L-ascorbic acid trisodium salt (Sigma-Aldrich, Steinheim, Germany)] for 24 h in an incubator (37°C, 5% CO2, 95% humidity). Afterwards, the explants were traumatized and cultivated up to 7 days in serum-free medium [DMEM, 1% sodium pyruvate, 0.5% L-glutamine, 1% non-essential amino acids, 0.5% penicillin/streptomycin, 10 μg/ml 2-phospho-L-ascorbic acid trisodium salt and 0.1% insulin-transferrin-selenium (ITS) (Sigma-Aldrich)]. All medium components were purchased from Biochrom (Berlin, Germany) unless specified otherwise.

### 2.2 Impact loading and statin treatment

Cartilage explants were subjected to a single impact load of 0.59 J using a drop-tower model as previously described (Riegger et al., 2016; Riegger et al., 2018b; Riegger and Brenner, 2019). Unloaded explants served as controls. Impacted cartilage explants were cultivated without further treatment or with either simvastatin or fluvastatin (Sigma-Aldrich) at different concentrations (1 μM, 5 and 10 µM) for 7 days. Fresh additives were added concomitantly with medium change every 2–3 days.

Simvastatin was activated by opening its lactone ring. In brief, 5 mg of simvastatin was dissolved in 125 µL of ethanol and 187.5 µl of 0.1 N NaOH. This solution was incubated at 50°C for 2 h and pH was afterwards adjusted to 7. Fluvastatin needs no activation and was directly dissolved in distilled water.

### 2.3 mRNA isolation and cDNA synthesis

For total RNA isolation, cartilage explants were snap frozen in liquid nitrogen 7 days after trauma and pulverized with a microdismembrator S (B. Braun Biotech, Melsungen, Germany). Subsequently, RNA was isolated using the Lipid Tissue Mini Kit (Qiagen, Hilden, Germany). RNA was reverse transcribed with the Omniscript RT Kit (Qiagen) and used for quantitative real-time PCR analysis (StepOnePlus™ Real-Time PCR System, Applied Biosystems, Darmstadt, Germany).

### 2.4 Quantitative real-time polymerase chain reaction (qRT-PCR)

Determination of the relative expression levels was performed by means of qRT-PCR analysis (2^−ΔΔCt^ method). To detect desired sequences, TaqMan^®^ Gene Expression Master Mix for TaqMan^®^ Gene Expression Assay (both Applied Biosystems) was used for following probes: Hs00264051_m1 (COL2A1), Hs00166657_m1, Hs02800695_m1 (HPRT1), Hs00233992_m1 (MMP-13), Hs00166163_m1 (NOX2/CYBB), Hs00418351_m1 (NOX4) and Hs00167309_m1 (SOD2). Power SYBR^®^ Green PCR Master Mix (Applied Biosystems) was used for 18S rRNA, 5′-CGC​AGC​TAG​GAA​TAA​TGG​AAT​AGG-3’ (forward) and 5′-CAT​GGC​CTC​AGT​TCC​GAA​A-3’ (reverse), and Platinum^®^ SYBR^®^ Green qPCR SuperMix-UDG (Invitrogen, Darmstadt, Germany) for GAPDH, 5′-TGG​TAT​CGT​GGA​AGG​ACT​CAT​G-3’ (forward) and 5′-TCT​TCT​GGG​TGG​CAG​TGA​TG-3’ (reverse). mRNA expression was determined by normalizing the expression levels separately to the endogenous controls (18S rRNA, GAPDH and HPRT1), and subsequently calculating the ratio mean values (2^-∆∆Ct^) in relation to the gene expression level of the untreated, unimpacted control.

### 2.5 Live/dead cell cytotoxicity assay

To determine the percentage of viable cells in untraumatized and traumatized cartilage explants with and without statin treatment, a Live/Dead^®^ Viability/Cytotoxicity Assay (Molecular Probes, Invitrogen) was performed. As previously described, unfixed tissue sections (0.5 mm thickness) were stained with 1 μM calcein AM and 2 μM ethidium homodimer-1 for 30 min (Riegger et al., 2016). After washing in phosphate buffered saline (PBS), they were microscopically analysed by means of a z-stack module (software AxioVision, Carl Zeiss, Jena, Germany). All cells on the image were counted manually (Software: ImageJ2 Version 2.3.0/1.53f). The average count per picture was about 500 cells/image (comprising dead and living cells).

### 2.6 Protein analysis by means of enzyme-linked immunosorbent assay (ELISA)

Quantity of biomarker release into culture media of cartilage explants was evaluated by means of ELISAs: secreted MMP-13 was determined using the Human Quantikine ELISA kit (RayBiotech, Norcross, GA, United States). Evaluation of collagen type II synthesis was performed using a collagen type II carboxy propeptide (CPII) ELISA (Ibex, Quebec, Canada). The assay quantifies CP II cleaved from procollagen II after its release into the matrix and directly correlates with newly synthetized collagen type II. Degradation of collagen type II was measured using a collagen type II cleavage (C2C) ELISA (Ibex), detecting a neoepitope generated during collagenase-mediated breakdown of collagen type II. The total amount of MMP-13, C2C and CP II, respectively, was relativized on the weight multiplied by cell viability of the corresponding cartilage explant as previously described (Riegger et al., 2018a; Riegger et al., 2016).

### 2.7 Gelatin zymography

Quantiﬁcation of pro-MMP-2 and active MMP-2 was performed by gelatin zymography as previously described (Riegger et al., 2016). In short, culture media from the cartilage explant culture were mixed 1:2 with nonreducing zymogram sample buffer (Bio-Rad, Munich, Germany), loaded onto 10% polyacrylamide gels (Carl Roth, Karlsruhe, Germany) containing 2 mg/ml gelatin (Merck, Darmstadt, Deutschland). After electrophoresis (Mini-PROTEAN Tetra Cell System, Bio-Rad) the gels were washed in zymogram renaturation buffer twice for 15 min and incubated in zymogram development buffer for 20 h at 37°C (both Bio-Rad). Staining with coomassie solution and subsequent destaining revealed clear bands originating from MMP activity. Band intensities (INT×mm^2^) were quantiﬁed with the Geldoc XR system (Bio-Rad) and relativized on weight and cell viability as mentioned above.

### 2.8 Isolation and cultivation of human primary chondrocytes

Chondrocytes were isolated from human cartilage (see above) by enzymatic digestion. In short, full-thickness cartilage was minced and digested for 45 min with 0.2% pronase (Sigma-Aldrich) and overnight with 0.025% collagenase (Sigma-Aldrich). After washing with PBS and filtration through a 40 μm cell strainer, cells were cultured in serum-containing chondrocyte medium (see above). Chondrocytes were split at a confluence of 80% and used at passage 4 and 5.

### 2.9 Chondrogenic redifferentiation of isolated chondrocytes

It is well known that chondrocytes lose their chondrogenic characteristics during *in vitro* expansion and that this is a reversible process (Riegger et al., 2016). Therefore, redifferentiation of dedifferentiated chondrocytes can be used as proof-of-principle experiments to investigate potential anti- or pro-chondroanabolic effects of therapeutics. For chondrogenic redifferentiation, 3.5 × 10^5^ chondrocytes at passage 4 or 5 were pelleted by centrifugation and cultivated as pellet culture for 28 days in chondrogenic differentiation medium (CDM) [DMEM supplemented with 4.5 g/L glucose and Ham’s F12 (1:1), 100 U/mL penicillin/streptomycin, 40 ng/mL L-proline, 0.1 μM dexamethasone, 50 μg/ml 2-phospho-L-ascorbic acid trisodium salt, 0.1% ITS (Sigma-Aldrich), 10 ng/ml recombinant human transforming growth factor-beta 3 (rhTGF-β3) (R&D Systems, Minneapolis, MN, United States) and 10 ng/ml recombinant human bone morphogenic protein 6 (rhBMP6) (Peprotech, Hamburg, Germany)]. During the first week, TGF-β3 and BMP-6 were freshly added every day; afterwards–similarly to statins–concomitantly with medium change at least twice a week. Influence of statins in long-term administration (28 days) and short-term application with deprivation after 7 days were investigated (9 donors, mean age 65, range 53–75). Assessment of chondrogenic differentiation was performed by using a well-established scoring system as previously described (Riegger et al., 2018b). Scoring was done independently by two observers and the mean was used for further analysis.

Presence of apoptotic cells was additionally evaluated by means of a TUNEL staining (Promega, Walldorf, Germany) in accordance to the manufacturer’s instructions.

### 2.10 Histological and immunohistochemical analysis

For immunohistochemistry (IHC), 3.5 μm-thick paraffin-embedded sections of the pellets from the chondrogenic redifferentiation were dewaxed, rehydrated and pre-digested for 30 min at 37°C for antigen retrieval with pepsin (1 mg/ml in 0.5 M acetic acid) (porcine gastric mucosa) (Sigma Aldrich), in case of collagen type II staining. Sections were treated with 3% hydrogen peroxide before starting the staining with the Dako labeled streptavidin-biotin (LSAB) 2 system-horseradish peroxidase (HRP) kit (Dako) and antibodies against collagen type II (AF5710; Acris, Hiddenhausen, Germany, and MA5-13026; Invitrogen). For histological staining of proteoglycans, Safranin O (Fisher Scientific) was used. In all samples, a final staining of cell nuclei by Gill’s haematoxylin No. 3 (Sigma-Aldrich) was performed.

### 2.11 Statistical analysis

Experiments were analysed using GraphPad Prism version 8.0 (GraphPad Software). Data sets with n ≥ 5 were tested for outliers with the Grubbs outlier test. Outliers were not included in statistical analyses. For parametric data sets, a one-way analysis of variance (ANOVA) with Sidak’s post-test was used. Nonparametric data sets were analysed by a Kruskal–Wallis test with Dunn’s post-test. Significant level was set to *α* = 0.05. Values in diagrams are given as boxplots (median; whiskers: min to max).

## 3 Results

### 3.1 Statins reduce cell death after cartilage trauma

**FIGURE 1:**
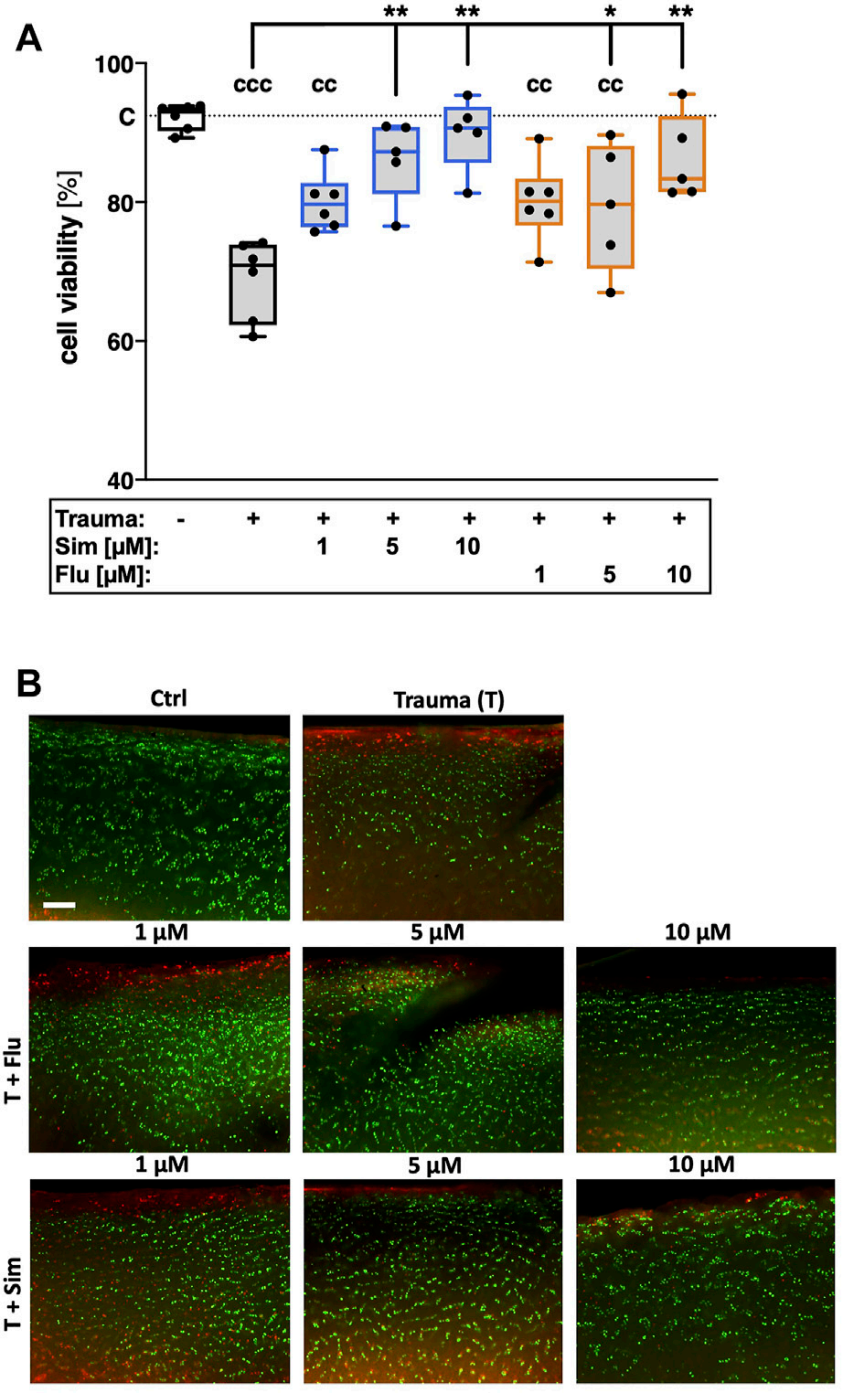
Effects of statins on cell viability after cartilage trauma. Cartilage explants were traumatized and treated with simvastatin (Sim) or fluvastatin (Flu) at different concentrations (1 μM, 5 and 10 µM) for 7 days **(A)** Cell viability was assessed by means of a live/dead staining **(B)** Exemplary fluorescence images; living cells appear in green while dead cells can be distinguished by red fluorescence. Significant differences between groups were depicted as [versus T] * = *p* < 0.05, ** = *p* < 0.01 [versus C] cc = *p* < 0.01, ccc = *p* < 0.001; *n* ≥ 5. Blank box = unimpacted control (C), shaded boxes = traumatized cartilage explants (T).

7 days after cartilage trauma, cell viability was decreased by 22% as compared to the non-impacted control tissue ([vs. C] *p* < 0.0001; Figure 1). Both statins significantly increased cell viability in traumatized cartilage explants in a concentration-dependent manner ([vs. T] 1 µM Sim: +8.8%; Flu: 9.2%; 5 µM Sim: +16.4%, *p* < 0.0001; Flu: +8.8%, *p* = 0.04; 10 µM Sim: +19.8%; Flu: +12.5%, both *p* < 0.0001). Considering the median values, treatment with simvastatin had higher cell protective effects as compared to fluvastatin.

### 3.2 Statins attenuate trauma-induced expression of MMP-13 and subsequent breakdown of collagen type II

**FIGURE 2:**
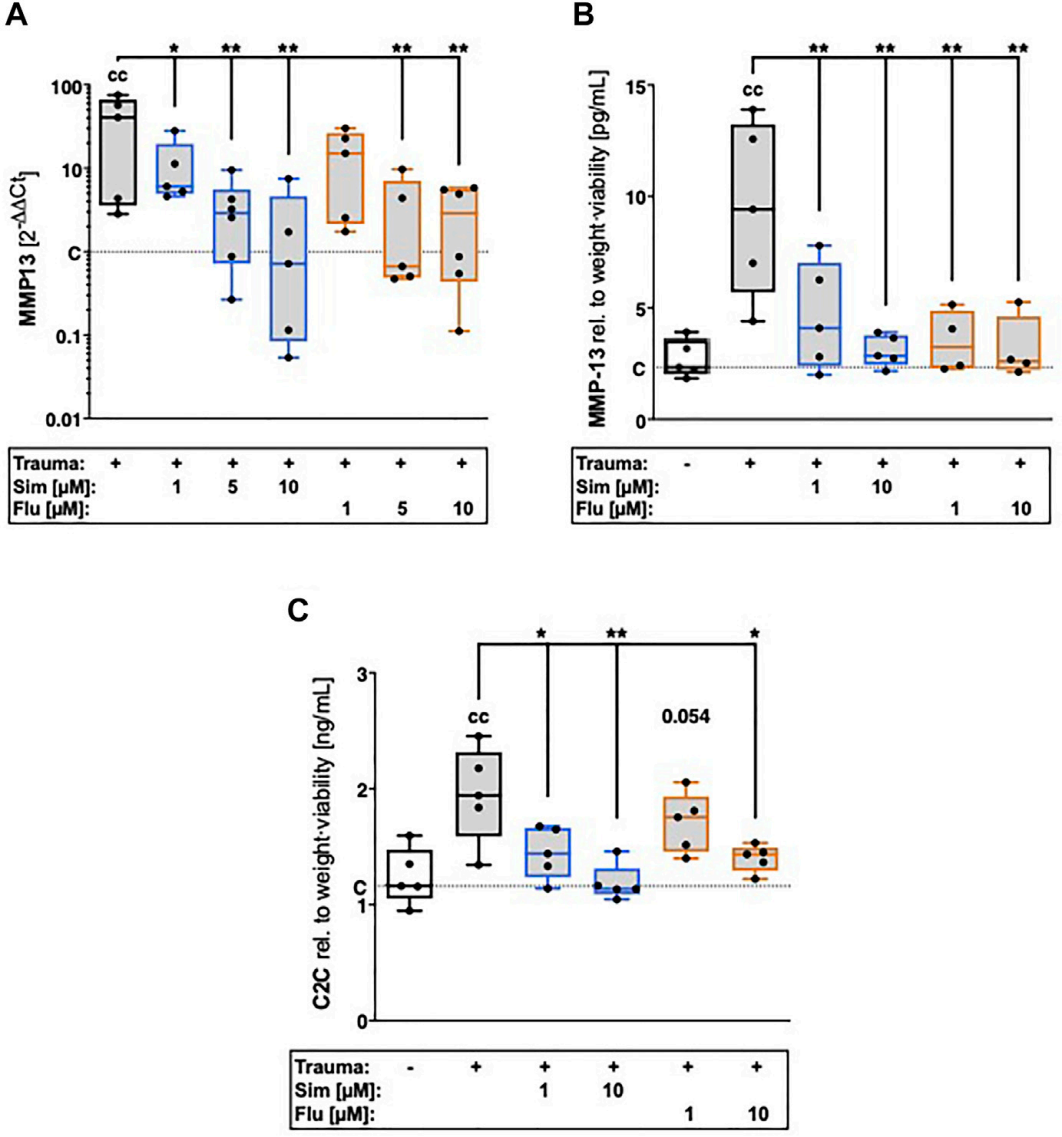
Effects of statin treatment on trauma-induced expression of catabolic enzymes and collagen type II breakdown. Catabolic processes were assessed by means of gene expression analysis of **(A)** MMP13 as well as **(B)** quantification of MMP-13 release and **(C)** collagen type II breakdown product C2C. Significant differences between groups were depicted as [versus T] * = *p* < 0.05, ** = *p* < 0.01 [versus C] c = *p* < 0.05, cc = *p* < 0.01; *n* ≥ 4. Blank box = unimpacted control (C), shaded boxes = traumatized cartilage explants (T).

Gene expression of MMP-13 was significantly enhanced 7 days after cartilage trauma ([vs. C] MMP13: 40.6-fold, *p* = 0.0008; Figure 2A). Increase of catabolic processes was also demonstrated by excessive MMP-13 release ([vs. C] +7.1 pg/ml; *p* = 0.0003; Figure 2B) and subsequent breakdown of collagen type II ([vs. C] + 0.8 ng/ml; *p* = 0.0013; Figure 2C). Statin therapy suppressed trauma-induced gene expression of MMP-13 ([vs. T] both statins 10 µM: 41.4-fold, *p* = 0.001) as well as MMP-13 secretion and subsequent breakdown of collagen type II, in a concentration-dependent manner. At a concentration of 10 μM, simvastatin reduced MMP-13 secretion by 70% ([vs. T] *p* = 0.0005) and generation of C2C by 41% ([vs. T] *p* = 0.0005), while fluvastatin attenuated MMP-13 secretion about 72% ([vs. T] *p* = 0.001) and generation of C2C by 26% ([vs. T] *p* = 0.016).

### 3.3 Statins suppress trauma-induced secretion and activation of MMP-2

**FIGURE 3:**
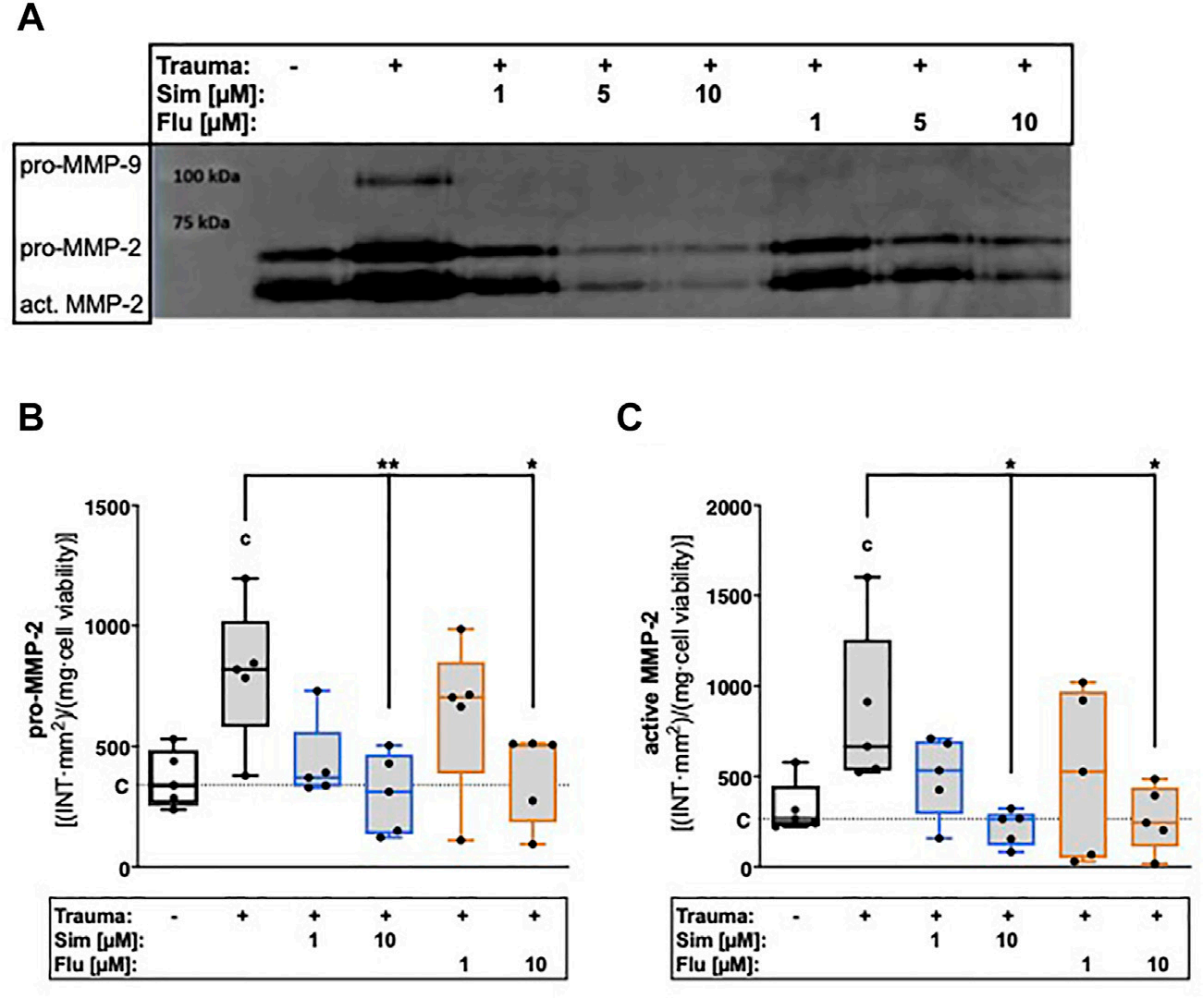
Effects of statin treatment on trauma-induced secretion and activity of gelatinases. Release of gelatinases MMP-2 and MMP-9 were measured by means of gelatin zymography as exemplarily demonstrated in **(A)**. Corresponding statistics of zymographically detectable amounts of **(B)** latent (pro-) MMP-2 and **(C)** active MMP-2. Significant differences between groups were depicted as [versus T] * = *p* < 0.05; ** = *p* < 0.01 [versus C] c = *p* < 0.05; n = 5. Blank box = unimpacted control (C), shaded boxes = traumatized cartilage explants (T).

Secretion of latent MMP-2 and presence of activated MMP-2, respectively, were 2.2- and 2.6-fold increased after trauma (Figures 3A–C). Treatment with high concentration (10 µM) of sim- or fluvastatin significantly suppressed the zymographically detectable amounts of latent ([vs. T] Sim: 2.6-fold, *p* = 0.007; Flu: 2.1-fold, *p* = 0.02) and active MMP-2 ([vs. T] Sim: 3.9-fold, *p* = 0.01; Flu: 3.2-fold, *p* = 0.02) within the supernatants of traumatized cartilage explants.

### 3.4 Statin treatment has differential effects on collagen type II expression

**FIGURE 4:**
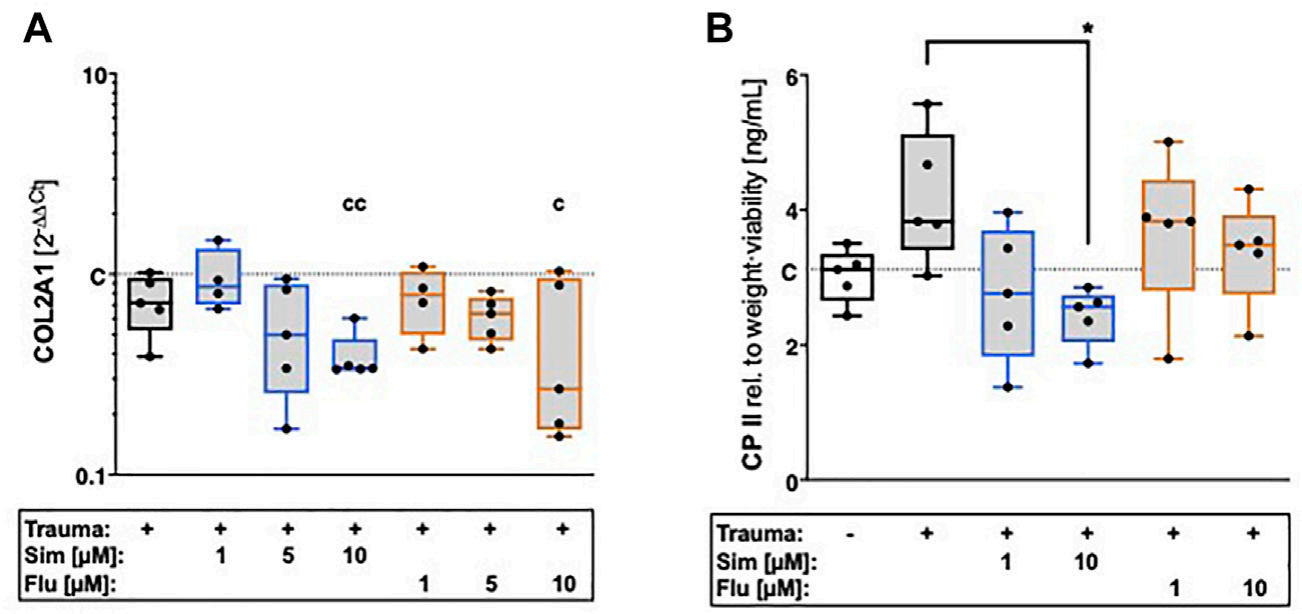
Effects of statin treatment on collagen type II expression after cartilage trauma. Expression of COL2 was determined by means of **(A)** gene expression analysis of COL2A1 as well as **(B)** quantification of CPII release. Significant differences between groups were depicted as [versus T] * = *p* < 0.05 [versus C] c = *p* < 0.05, cc = *p* < 0.01; *n* = 5. Blank box = unimpacted control (C), shaded boxes = traumatized cartilage explants (T).

After cartilage trauma, gene expression of collagen type II was rather reduced as compared to unimpacted control, though not significantly different (Figure 4A). Simvastatin further suppressed the gene expression of COL2A1 ([vs. C] -66%, *p* = 0.003) as well as collagen type II synthesis ([vs. T] -18% *p* = 0.012) in a concentration-dependent manner. Although fluvastatin suppressed the gene expression of COL2A1 ([vs. C] -73%, *p* = 0.02), no influence was found on protein level.

### 3.5 Statin treatment reduces trauma-induced gene expression of NADPH oxidases and enhances that of SOD2

**FIGURE 5:**
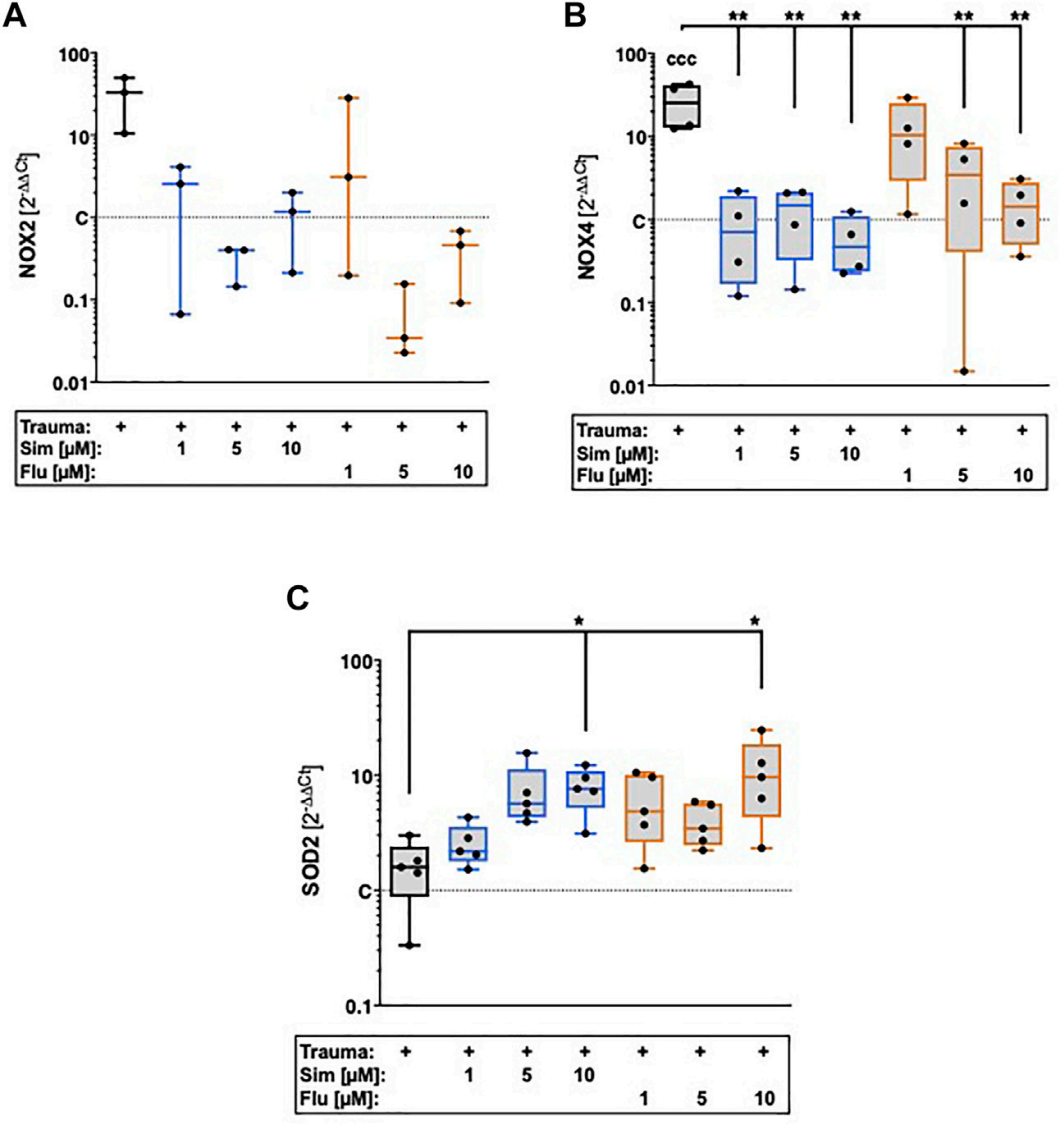
Effects of statin treatment on gene expression of NADPH oxidases and SOD2 after cartilage trauma. Potential effects of statins on intracellular oxidative stress were determined by means of gene expression analysis of **(A)** NOX2 **(B)** NOX4 and **(C)** SOD2. Significant differences between groups were depicted as [versus T] * = *p* < 0.05, ** = *p* < 0.01 [versus C] ccc = *p* < 0.001; *n* ≥ 3. Shaded boxes = traumatized cartilage explants (T).

Influence of statin treatment on intracellular oxidative stress after cartilage trauma was determined by gene expression of NADPH oxidase 2 (NOX2) and NOX4 as well as mitochondrial superoxide dismutase 2 (SOD2). Although the gene expression of NOX2 was increased after trauma, the expression levels were generally low and could only be detected in 3 of 6 donors (Figure 5A). However, statin therapy noticeably reduced the gene expression of NOX2. Cartilage trauma also induced the gene expression of NOX4 ([vs. C] 25.6-fold, *p* = 0.0003), which could be significantly attenuated by statin treatment (Figure 5B). While simvastatin suppressed the gene expression of NOX4 at all concentrations, effects of fluvastatin were clearly concentration-dependent. In contrast to NOX2 and NOX4, gene expression of SOD2 was significantly enhanced by both simvastatin and fluvastatin treatment at a concentration of 10 µM ([vs. T] Sim: 4.8-fold, *p* = 0.03; Flu: 6-fold, *p* = 0.02) (Figure 5C).

### 3.6 Long- and short-term application of statin treatment has contrary effects on chondrogenic redifferentiation of isolated chondrocytes

**FIGURE 6:**
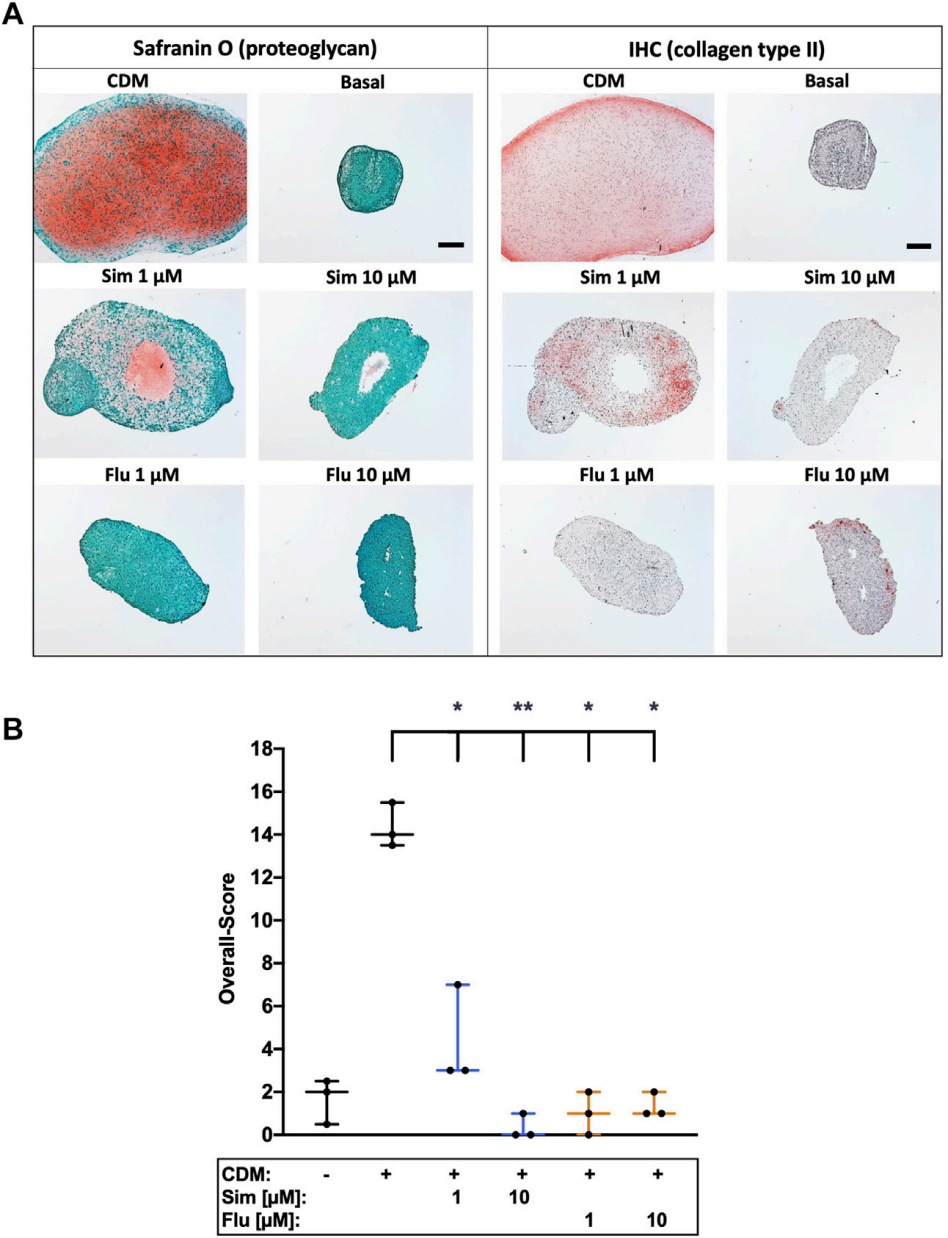
Effects of long-term statin treatment on chondrogenic differentiation of isolated chondrocytes **(A)** Chondrogenic redifferentiation of high passage chondrocytes was determined by means of proteoglycan (Safranin O) staining and collagen type II (IHC), as exemplarily demonstrated **(B)** Neocartilage formation was further assessed using a defined scoring system. Black scale bar = 200 μm; Significant differences between groups were depicted as [versus CDM] * = *p* < 0.05; ** = *p* < 0.01; *n* = 3.

**FIGURE 7:**
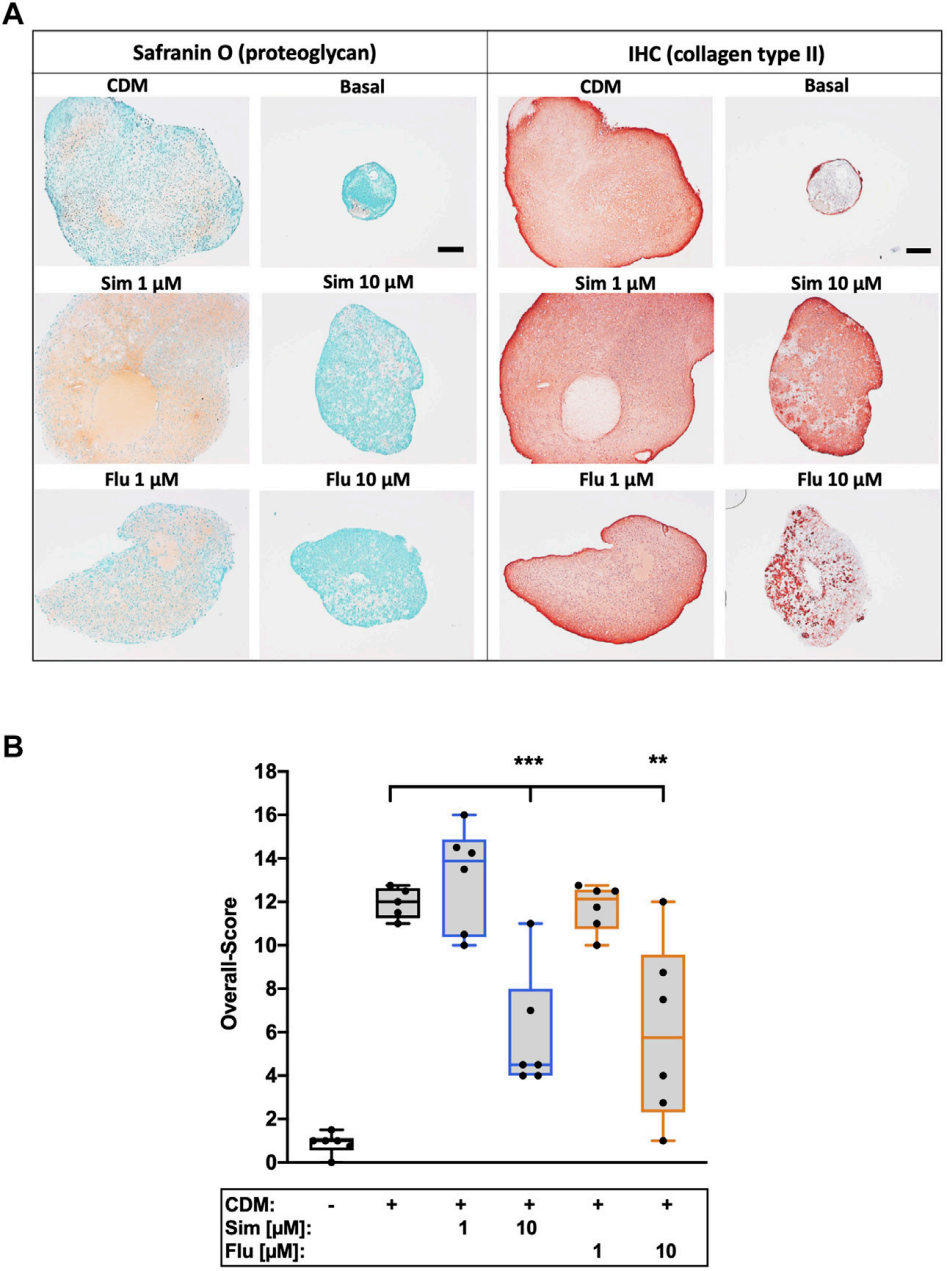
Effects of short-term application of statins on chondrogenic differentiation of isolated chondrocytes **(A)** Exemplarily demonstrated the staining of proteoglycan (Safranin O) and collagen type II (IHC) of the redifferentiation of high passage chondrocytes **(B)** Chondrogenic redifferentiation was assessed by a defined scoring system. Black scale bar = 200 μm; n = 6. Significant differences between groups were depicted as [versus CDM] ** = *p* < 0.01; *** = *p* < 0.001.

Influence of statins on chondroanabolism and stabilization of the chondrogenic phenotype, respectively, was determined by redifferentiation of high passage chondrocytes in presence of simvastatin or fluvastatin. As compared to the unstimulated control (basal medium), 3D cultivation of high passage chondrocytes in CDM for 28 days significantly enhanced the expression of ECM compounds as demonstrated for proteoglycan (Safranin O staining) and collagen type II. Long-term co-stimulation with low (1 µM) or high (10 µM) concentrations of statins during cultivation significantly impaired the chondrogenic redifferentiation (Figure 6A). Previously we investigated anti-chondroanabolic effects of N-acetyl cysteine (NAC) which could be reversed after deprivation of the agent (Riegger et al., 2018a). Therefore, we investigated short-term application of statins during chondrogenic redifferentiation with deprivation after 7 days and similar reversible effects with statins (l µM) could be observed. Short-term application exhibited no inhibitory effects on redifferentiation at low concentrations. Nevertheless, high concentrations still impaired the redifferentiation significantly even after deprivation (Figure 7A). Semi-quantitative assessment of the chondrogenic differentiation and neocartilage formation, respectively, was performed in a modified scoring system, considering proteoglycan and collagen type II staining intensities as well as pellet size, ECM formation (cell-cell distance) and cell morphology (Riegger et al., 2018b). Continuous application of statins led to a clear reduction in all scoring criteria (Supplementary Table S1), resulting in significantly lower overall scores (Figure 6B). However, deprivation of statins reversed inhibitory effects, reflected in an increase of the overall scores. In addition, for simvastatin at low concentrations, a clearly stronger Safranin O staining was apparent compared to the CDM control (Figure 7B) (Supplementary Table S2).

Furthermore, we observed an enhanced number of apoptotic cells after chondrogenic redifferentiation by means of exemplary TUNEL staining in particular after continuous application of statins. This effect was clearly reduced after short-term treatment with the drugs, but still increased as compared to the positive control (Supplementary Figure S3).

## 4 Discussion

For many years, statin administration has been discussed as potential therapeutic approach in OA disease, though with controversial results regarding both clinical and experimental studies. While many reports provide clear evidence of immunomodulatory effects of statin therapy, resulting in alleviated synovial inflammation in RA (Greenwood et al., 2006; McCarey et al., 2004; Leung et al., 2003), contradictory outcomes have been observed regarding statin influence on the chondroanabolic phenotype of chondrocytes and MSC, which might have detrimental effect on cartilage integrity (Fafilek et al., 2017; Hatano et al., 2003; Han and Kim, 2016; Zhang et al., 2019; Izadpanah et al., 2015). Taken together, appropriate *ex vivo* experiments scrutinizing the impact of statin application on cartilage homeostasis are rare.

Our *ex vivo* cartilage trauma model allows investigation of mechanically-induced pathomechanisms in human cartilage without influence of synovium-derived pro-inflammatory and catabolic mediators. However, the model includes a low-grade inflammatory response from the cartilage cells, including release of nitric oxide, prostaglandin E2 and D2, as well as low levels of interleukin-6 (IL-6), but no detectable amounts of tumor necrosis factor (TNF) and interleukin-1 beta (IL-1B) (Riegger and Brenner, 2019; Joos et al., 2011). Here, we evaluated possible therapeutic effects of statins after cartilage injury focusing on cell death and chondrocyte metabolism, including the chondrogenic redifferentiation of high passage human chondrocytes.

Previous studies on trauma-related cell death in cartilage revealed, that chondrocytes undergo both necrotic and apoptotic cell death. While apoptosis is thought to be triggered by posttraumatic pathomechanisms, including oxidative stress, and contributes to ongoing cell loss, necrosis might be rather ascribed to the direct mechanical loading (Riegger et al., 2016). Statins have been shown to regulate pro- and anti-apoptotic processes by differently influencing the expression of Bcl-2 family members (Wood et al., 2013). However, statin therapy was described as predominately cell protective against injury-related and oxidative stress-induced cell death *in vivo* and *in vitro* (Xu et al., 2008; Zhao et al., 2012; Rajtík et al., 2012; Obata, 2006). In fact, both simvastatin and fluvastatin were reported to have antioxidant activity and attenuated H_2_O_2_-induced cell death (Xu et al., 2008; Zhao et al., 2012). In that regard, simvastatin was most effective against hydroxyl radicals, while fluvastatin efficiently reduced peroxyl radicals (Franzoni et al., 2003). Our results support these findings by demonstrating enhanced gene expression levels of SOD2 as well as simultaneous suppression of trauma-induced NOX2 and NOX4 in statin-treated cartilage. Beyond that, Hsieh et al. described simvastatin-mediated maintenance of mitochondrial function and promotion of both autophagy and mitophagy in cardiomyocytes, implying mitoprotective effects (Hsieh et al., 2019). Mitoprotective therapy can be considered as a sort of specific antioxidative approach, targeting mitochondrial dysfunction. Since mitochondrial stress represents a crucial aspect in post-injurious cartilage degeneration and chondrocyte death, mitoprotection turned out to be a promising therapeutic approach after cartilage trauma (Riegger and Brenner, 2020; Bartell et al., 2020). Taken together, cell protective effects observed after cartilage trauma in this study might result from antioxidative and mitoprotective effects of statin therapy. However, in line with previous reports, which described not only anti- but also pro-apoptotic effects of statins, we observed an enhanced number of TUNEL-positive cells during chondrogenic redifferentiation in presence of statins (Wood et al., 2013). This implicates that the cell protective effects of statins in chondrocytes might depend on the differentiation state, which is associated with the cellular metabolism and considerably differs between immature and mature chondrocytes.

In contrast, statins were found to induce mitochondrial dysfunction in muscle cells, leading to statin-associated muscle disease (SAMS), the most common side effect caused by statin use, though, the mechanisms have not been completely unraveled so far (Ramachandran and Wierzbicki, 2017). Moreover, high concentrations of statins largely suppressed proliferation and enhanced cell death in isolated chondrocytes (Fafilek et al., 2017; Chang et al., 2014) as well as other cell types (Wood et al., 2013). Although we could confirm enhanced cytotoxicity of simvastatin and fluvastatin on isolated human cartilage cells, occurring in a time and concentration-dependent manner (data not shown), our data rather indicate mitoprotective effects of statins as described above. Mullen et al. hypothesized that susceptibility to statin-induced cytotoxicity is based upon insulin-like growth factor-1 (IGF-1)/Akt signaling and subsequent Akt phosphorylation. Consequently, disruption of IGF-1/Akt signaling resulted in statin-mediated mitochondrial dysfunction, while its integrity provided cell protection (Mullen et al., 2011). Although, IGF-1/Akt signaling is known to play a crucial role in chondrocytes, comprising maintenance of the chondrogenic phenotype and suppression of apoptosis (Oh and Chun, 2003), further studies are needed to confirm an association between statin-related cytotoxicity and alteration of IGF-1/Akt signaling in isolated cartilage cells.

Statin therapy exhibited anti-anabolic effects as we observed in collagen type II expression after cartilage trauma and impaired ECM synthesis during chondrogenic redifferentiation of high passage chondrocytes in a long-term application. Interestingly, studies reporting chondroanabolic properties of statins only considered a short observation period (≤72 h) (Fafilek et al., 2017; Hatano et al., 2003; Han and Kim, 2016; Sverdrup et al., 2010; Yu and Kim, 2018; Goto et al., 2017), while long-term stimulation (≥7 days) with statins, comparable to the present study, usually had no beneficial effect or even resulted in impaired expression of chondroanabolic markers (Hatano et al., 2003; Izadpanah et al., 2015; Goto et al., 2017). In fact, it has been described that certain amounts of MMPs and reactive oxygen species are required for chondrogenic differentiation; consequently, its elimination impairs the differentiation process (Li and Dong, 2016). In line with these results, we found that statin therapy significantly suppressed the gene expression of NADPH oxidases NOX2 and NOX4, which were described as essential during chondrogenic differentiation (Ki et al., 2010). Although Zhang et al. demonstrated that long-term administration of simvastatin at very low dosages ≤0.1 µM promoted chondrogenic differentiation (Zhang et al., 2019), higher concentrations (≥5 µM) might be needed to achieve anti-catabolic and thus chondroprotective effects, as our data imply. Previously, we faced a similar dilemma regarding the antioxidant NAC, which had cell- and chondroprotective effects after cartilage trauma, but suppressed chondroanabolic events, most likely due to its strong antioxidative potency and inhibition of MMPs (Riegger et al., 2018b; Riegger et al., 2018a). Our *in vivo* experiments using NAC after cartilage injury confirmed that initial harm reduction might all in all be more important than possible impairment of ECM synthesis during therapeutic application, which also seems to be true for statin therapy (Akasaki et al., 2009; Goto et al., 2017; Riegger et al., 2019). Interestingly, deprivation of statins, applied at a low dosage, was associated with an increase of ECM synthesis compared with continuous application. Simvastatin displayed a slight increase in the overall chondrogenic differentiation score compared to the CDM control, though this was not significant. Our effects are also consistent with further studies: Terabe et al. reported that simvastatin enhances SOX9 as well as COL2A expression in human articular cartilage derived from OA patients. Furthermore, in bovine articular chondrocytes simvastatin enhanced the BMP2 expression (Terabe et al., 2019). SOX-9 is known as a driving transcription factor promoting chondrogenic differentiation with simultaneous inhibition of differentiation into hypertrophic chondrocytes (Li and Dong, 2016). BMP-2 stabilizes the chondrogenic phenotype and therefore prevents chondrocyte dedifferentiation, resulting in an increased synthesis of collagen type II (Demoor et al., 2014).

Comparable to NAC, antioxidative potential of statins might also play an important role in attenuation of trauma-induced catabolic processes (Riegger et al., 2016). In fact, we observed enhanced expression of SOD2 by statin therapy, which might stabilize mitochondrial function after cartilage trauma and thus reduce catabolic expression (Koike et al., 2015). Moreover, anti-catabolic and chondroprotective effects of statins in IL-1B stimulated chondrocytes and cartilage, respectively, have been ascribed to the inhibition of mevalonate synthesis and subsequent protein geranylgeranylation, i.e. of Ras and Rho GTPase, usually resulting in nuclear factor-kappa B (NF-kB) as well as c-Jun N-terminal kinase (JNK) and p38 mitogen-activated protein kinases (p38 MAPK) pathway activation (Sverdrup et al., 2010; Abeles and Pillinger, 2006; Barter et al., 2010). While Sverdrup et al. suggested that RhoA geranylgeranylation was not directly involved in this process (Sverdrup et al., 2010), other sources provide clear evidence of statin-mediated inhibition of RhoA signaling (Oh and Chun, 2003).

To our knowledge, this is the first proof-of-principle study confirming the potential efficacy of statin therapy after cartilage trauma using a human *ex vivo* model. Our results imply significant cell and chondroprotective effects of simvastatin and fluvastatin, but also anti-chondroanabolic feature during long-term treatment. We therefore assume that a finely tuned administration, considering both statin concentration and duration of its application, plays a crucial role in promoting chondroanabolism and still achieving cell protection, as well as antioxidative and anti-catabolic effects. Therefore, statin therapy might be beneficial during the acute phase after joint injury by reducing the risk of ongoing cartilage degeneration and development of PTOA.

## Data Availability

The original contributions presented in the study are included in the article/Supplementary Material, further inquiries can be directed to the corresponding author.

## Abbreviations

ANOVA, analysis of variance; ADAMTs, a disintegrin and metalloproteinase with a thrombospondin motif; CDM, chondrogenic differentiation medium; CPII, collagen type II carboxy propeptide; C2C, collagen type II cleavage; DMEM, Dulbecco’s Modified Eagle’s Medium; ECM, extracellular matrix; Flu, fluvastatin; HMG-CoA, hydroxymethylglutaryl-coenzyme A; HRP, horseradish peroxidase; IHC, immunohistochemistry; INT, intensity; ITS, insulin-transferrin-selenium; IL, interleukin; LSAB2, labeled streptavidin-biotin 2 system; MMP, matrix metalloproteinases; MSC, mesenchymal stem cells; NAC, N-acetyl cysteine; NOX, NADPH oxidase; OA, osteoarthritis; PBS, phosphate buffered saline; PTOA, post-traumatic osteoarthritis; Sim, simvastatin; RA, rheumatoid arthritis; rhTGF-β3, recombinant human transforming growth factor-beta 3; rhBMP6, recombinant human bone morphogenic protein 6; SAMS, statin-associated muscle disease; TNF, tumor necrosis factor.
