# Supplementary material for: Simvastatin and fluvastatin attenuate trauma-induced cell death and catabolism in human cartilage
Source: Front Bioeng Biotechnol. 2022 Sep 9;10:965302. doi: 10.3389/fbioe.2022.965302 (PMC9500391; doi:10.3389/fbioe.2022.965302)

Supplementary Table S1. Assessment of human chondrocytes (passage 4) after chondrogenic differentiation for 28 d with continuous statin treatment for 28 d. Results of individual categories given as mean ± standard deviation; n = 3. Basal= chondrocyte culture medium (10% FCS); CDM= chondrogenic differentiation medium; Sim= simvastatin; Flu= fluvastatin.

Approach	Diameter	Collagen II	Proteoglycan (Safranin O)	Matrix produced	Cell morphology
Basal	0.33 ± 0.58	0.0	0.33 ± 0.58	0.33 ± 0.58	0.42 ± 0.49
CDM	2.17 ± 0.29	3.33 ± 0.58	3.83 ± 0.29	3.42 ± 0.49	2.92 ± 1.02
Sim 1 µM	0.83 ± 0.29	1.5 ± 0.87	0.5 ± 0.87	0.83 ± 0.82	0.42 ± 0.49
Sim 10 µM	0.33 ± 0.58	0.0	0.0	0.0	0.0
Flu 1 µM	0.0	0.67 ± 1.16	0.0	0.17 ± 0.41	0.0
Flu 10 µM	0.67 ± 0.58	0.0	0.0	0.33 ± 0.52	0.0

Supplementary Table S2. Assessment of human chondrocytes (passage 4) after chondrogenic differentiation for 28 d with short-term statin treatment for 7 d. Results of individual categories given as mean ± standard deviation; n = 6. Basal= chondrocyte culture medium (10% FCS); CDM= chondrogenic differentiation medium; Sim= simvastatin; Flu= fluvastatin.

Approach	Diameter	Collagen II	Proteoglycan (Safranin O)	Matrix produced	Cell morphology
Basal	0.0	0.63 ± 0.49	0.25 ± 0.42	0.0	0.0
CDM	1.00 ± 0.71	3.80 ± 0.27	2.80 ± 0.91	2.15 ± 0.49	2.20 ± 0.48
Sim 1 µM	1.50 ± 0.55	3.50 ± 1.00	3.50 ± 0.55	2.54 ± 0.60	2.08 ± 0.75
Sim 10 µM	0.67 ± 0.52	2.75 ± 0.82	1.04 ± 1.01	0.92 ± 0.79	0.46 ± 0.78
Flu 1 µM	1.17 ± 0.41	3.38 ± 0.70	2.75 ± 0.99	2.42 ± 0.26	2.04 ± 0.60
Flu 10 µM	0.50 ± 0.55	2.29 ± 1.19	1.29 ± 1.68	1.17 ± 0.75	0.75 ± 0.76

Supplementary Figure S3. Exemplary images of TUNEL staining after chondrogenic differentiation (28 d). Chondrocytes were differentiated under short-term (7 d) or continuous (28 d) statin treatment. Basal= chondrocyte culture medium (10% FCS), negative control; CDM= chondrogenic differentiation medium, positive control; Sim= simvastatin; Flu= fluvastatin.

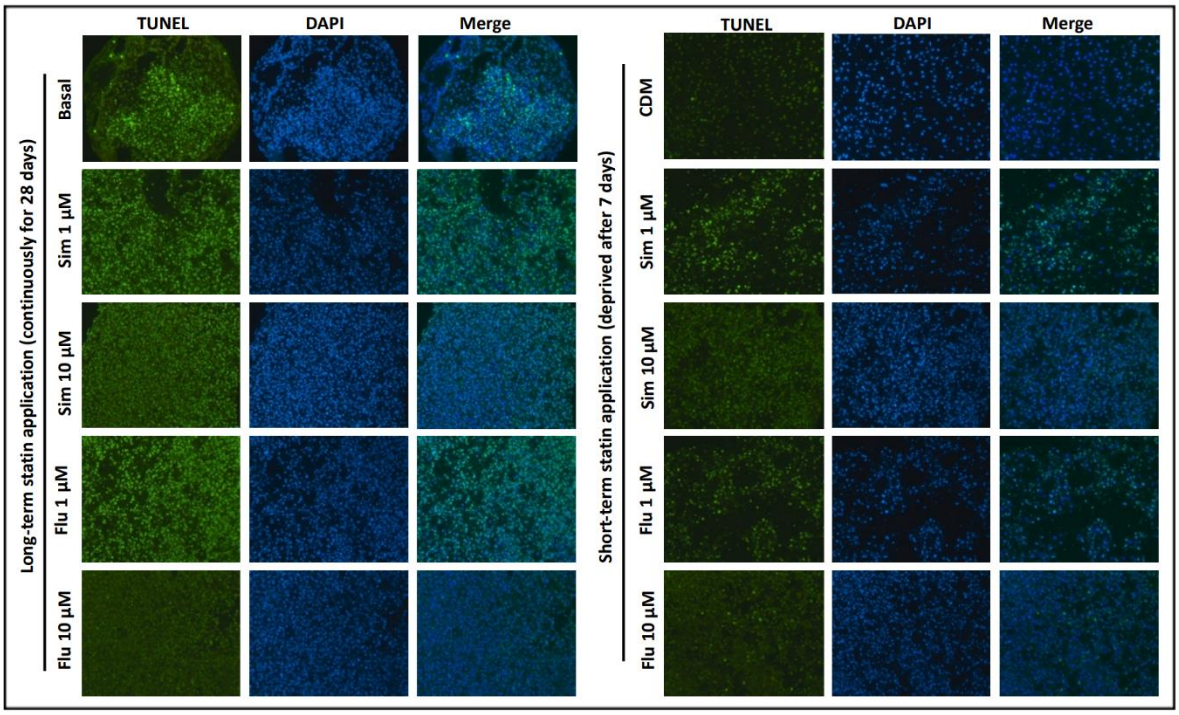

Supplement: Supplementary file 1 [file DataSheet1.pdf]
